# Adipose afferent reflex is enhanced by TNFα in paraventricular nucleus through NADPH oxidase-dependent ROS generation in obesity-related hypertensive rats

**DOI:** 10.1186/s12967-019-2006-0

**Published:** 2019-08-07

**Authors:** Lei Ding, Ying Kang, Hang-Bing Dai, Fang-Zheng Wang, Hong Zhou, Qing Gao, Xiao-Qing Xiong, Feng Zhang, Tian-Run Song, Yan Yuan, Ming Liu, Guo-Qing Zhu, Ye-Bo Zhou

**Affiliations:** 1 Department of Physiology, Nanjing Medical University, 101 Longmian Road, Nanjing, 211166 China; 2 Department of Pathophysiology, Xuzhou Medical University, Xuzhou, 221004 China

**Keywords:** Adipose afferent reflex, TNFα, Paraventricular nucleus, Sympathoexcitation, Obesity, Reactive oxygen species

## Abstract

**Background:**

The adipose afferent reflex (AAR), a sympatho-excitatory reflex, can promote the elevation of sympathetic nerve activity (SNA) and blood pressure (BP). Inflammation in the paraventricular nucleus (PVN) involves sympathetic abnormality in some cardiovascular diseases such as hypertension. This study was designed to explore the effects of tumor necrosis factor alpha (TNFα) in the PVN on the AAR and SNA in rats with obesity-related hypertension (OH) induced by a high-fat diet for 12 weeks.

**Methods:**

Renal sympathetic nerve activity (RSNA) and mean arterial pressure (MAP) were continuously recorded in anesthetized rats, and their responses to capsaicin (CAP) stimulation of the right inguinal white adipose tissue were used to evaluate the AAR.

**Results:**

Compared to the control rats, the systolic blood pressure (SBP), plasma norepinephrine (NE, indicating SNA) and TNFα levels, TNFα mRNA and protein levels, reactive oxygen species (ROS) content and NADPH oxidase activity in the PVN were significantly elevated in rats with OH. TNFα in the PVN markedly enhanced sympathoexcitation and AAR. Moreover, the enhancement of AAR caused by TNFα can be significantly strengthened by the pretreatment of diethyldithiocarbamate (DETC), a superoxide dismutase inhibitor, but attenuated by TNF-α receptor antagonist R-7050, superoxide scavenger PEG-SOD and NADPH oxidase inhibitor apocynin (Apo) in rats with OH. Acute microinjection of TNF-α into the PVN significantly increased the activity of NADPH oxidase and ROS levels in rats with OH, which were effectively blocked by R-7050. Furthermore, our results also showed that the increased levels of ROS, TNFα and NADPH oxidase subunits mRNA and protein in the PVN of rats with OH were significantly reversed by pentoxifylline (PTX, 30 mg/kg daily ip; in 10% ethanol) application, a cytokine blocker, for a period of 5 weeks. PTX administration also significantly decreased SBP, AAR and plasma NE levels in rats with OH.

**Conclusions:**

TNFα in the PVN modulates AAR and contributes to sympathoexcitation in OH possibly through NADPH oxidase-dependent ROS generation. TNFα blockade attenuates AAR and sympathoexcitation that unveils TNFα in the PVN may be a possible therapeutic target for the intervention of OH.

## Background

Worldwide prevalence of obesity has rapidly increased, especially in younger population in recent years and become a major threat to people’s health [1, 2]. Chronic sympathetic overactivation is a hallmark of obesity and contributes to the development of cardiovascular diseases including hypertension and heart failure [3–5]. Moreover, central mediation plays an important role in the enhancement of sympathoexcitation in obesity [6–8].

Recent studies on abnormality of sympathetic nerve activity (SNA) in obesity has targeted the hypothalamus and illustrated that hypothalamic paraventricular nuclei (PVN), an important central site for the integration of SNA and the regulation of cardiovascular function, can mediate obesity pathogenesis [9–12]. The increased abdominal visceral fat composed of white adipose tissue (WAT) is a very important contributing factor for the sympathetic overdrive and hypertension [13]. Neuroanatomical and functional studies indicate the existence of sensory and sympathetic innervations in the WAT [14, 15]. The adipose afferent reflex (AAR), a sympatho-excitatory reflex for increasing SNA and blood pressure (BP), can be induced by the stimulation of afferent nerve fibres of WAT with capsaicin (CAP) [13]. A related study from our laboratory demonstrated PVN mediated the enhanced AAR implicates the pathogenesis of obesity-related hypertension (OH) [16].

Increasing evidence show that inflammation is involved in the progression of many diseases, and it is a common feature both in obesity [17] and hypertension [18]. Elevated proinflammatory cytokines (PICs) are transported into the PVN primarily via the circumventricular organs [19], and the accumulated PICs such as tumor necrosis factor α (TNFα) in the PVN can regulate SNA and cardiovascular function in rats with hypertension or chronic heart failure (CHF) [20–22]. Moreover, the elevated PICs in the PVN induce the production of reactive oxygen species (ROS) which are cytotoxic, further contributing to sympathoexcitatory effects [23–26]. However, the increase of ROS induced by TNFα for the modulation of AAR and SNA in the PVN in OH is unexplored. Some studies also showed that the rats with CHF or hypertension had an increased level of TNFα or ROS in the PVN, resulting in the sympathoexcitation [21, 22]. TNFα as a contributor to oxidative stress, either directly or indirectly plays an important role in the progression of obesity [27]. TNFα modulates the activity and protein expression of NADPH oxidase, a potential source of ROS, involving the progress of cardiovascular diseases [21, 22]. However, the pathophysiological significance of the increased levels of TNFα within the PVN modulating AAR and sympathetic outflow in OH remains obscure. Therefore, the present study was designed to verify the hypothesis that TNFα may modulate NADPH oxidase-dependent ROS generation in the PVN involving the enhancement of AAR and SNA in rats with OH.

## Materials and methods

Adult male Sprague–Dawley rats weighing 300–350 g were used for the induction of OH rats by using high-fat diet (HFD, 45% kcal as fat: 45% fat, 40% carbohydrate, and 15% protein. TROPHIC Animal Feed High-tech Co Ltd, Nantong, China) for 12 weeks in this study. They were housed in a temperature and light-controlled room and provided with water and chow ad libitum. After 12 weeks, rats consuming the HFD were ranked based on weight gain and BP. The rats with more weight gains and BP ≥ 140 mmHg were referred to as OH group, and the rats with control diet (12% kcal as fat: 12% fat, 60% carbohydrate and 28% protein. TROPHIC Animal Feed High-tech Co Ltd, Nantong, China) were used as Control group. The experimental procedures were in compliance with the Guide for the Care and Use of Laboratory Animals (NIH publication, 8th edition, 2011) and approved by the Experimental Animal Care and Use Committee of Nanjing Medical University. The rats with OH were subsequently injected with PTX (30 mg/kg daily ip; in 10% ethanol) or vehicle (10% ethanol alone) for 5 weeks. Rats were killed under anesthesia with an overdose of sodium pentobarbital by intraperitoneal injection, and the plasma and PVN tissues were collected for further analysis.

### Measurement of body weight (BW) and systolic blood pressure (SBP)

The BW and SBP of rats were measured in a conscious state. SBP of tail artery was detected by using a non-invasive computerized tail-cuff system (NIBP, AD Instruments, Sydney, Australia). The rat was warmed for about 20 min to detect the pulsation of tail artery and obtain a steady pulse level. Moreover, in order to minimize the SBP fluctuation, the rat was trained for SBP measurement at least 10 days before the experiment formally started. This experiment was repeated 10 times in a rat, and SBP value was the mean of ten replicates.

### General procedures of acute experiment

Acute experiment was carried out after 12 or 17 weeks, the rats underwent anesthesia with urethane (800 mg/kg, ip) and α-chloralose (40 mg/kg, ip). Midline incisions were made for exposing the trachea and the right carotid artery, then the trachea was cannulated and connected with a rodent ventilator (Model 683, Harvard Apparatus Inc, USA), and the carotid artery was cannulated and connected with pressure transducer for recording of mean arterial pressure (MAP). A PowerLab data acquisition system (8/35, AD Instruments, Sydney, Australia) was used for simultaneous recording of the renal sympathetic nerve activity (RSNA) and MAP.

### RSNA recording

The dynamic alterations of sympathetic outflow was assessed by recording RSNA continuously. Simply, the retroperitoneal incision was made for the isolation of left renal sympathetic nerve which was cut distally to eliminate its afferent activity, then the nerve was placed on a pair of silver electrodes. An AC/DC differential amplifier (Model 3000; A-M System, Washington, DC, USA) was used to amplify the RSNA which was filtered with a band-pass between 10 and 3000 Hz, and the signals were integrated at a constant of 100 ms. At the end of experiment, the baseline noise was measured after section of the central end of the nerve, then subtracted from the integrated RSNA data. The RSNA change (in %) was the percent change from baseline RSNA value which were determined by averaging 2 min of its maximal responses after a certain chemical microinjection into the PVN. AAR was evaluated by averaging 2 min of the maximal RSNA and MAP responses to CAP within the time range from 10 to 30 min after the application of CAP, and the RSNA change (in %) was the percent change from values before CAP.

### AAR induction and evaluation

CAP, a common and valuable tool for exploring the function of sensory afferent fibres, was used to stimulate the sensory afferent fibres in right inguinal WAT (iWAT) for the induction of AAR. Briefly, the right iWAT area was exposed by an inguinal incision, and four thin and sharp stainless steel tubes (0.31 mm outer diameter) were inserted into iWAT (3 mm) under the surface of fat pads. The tips of these tubes were spaced (4 mm) apart from each other and connected with a 4-channel programmable pressure injector (PM2000B; MicroData Instrument, Plainfield, NJ, USA). CAP (1.0 nmol/μL) was injected into the right iWAT at a rate of 4.0 μL/min for 2 min for each site. The RSNA and MAP responses to injection of CAP were used to evaluate the AAR. At the end of experiment, Evans blue (8 μL) was injected into the iWAT for histological identification of WAT, and the diffusion diameter of blue dye localized in iWAT was less than 3 mm in all rats.

### PVN microinjection

**Fig. 1:**
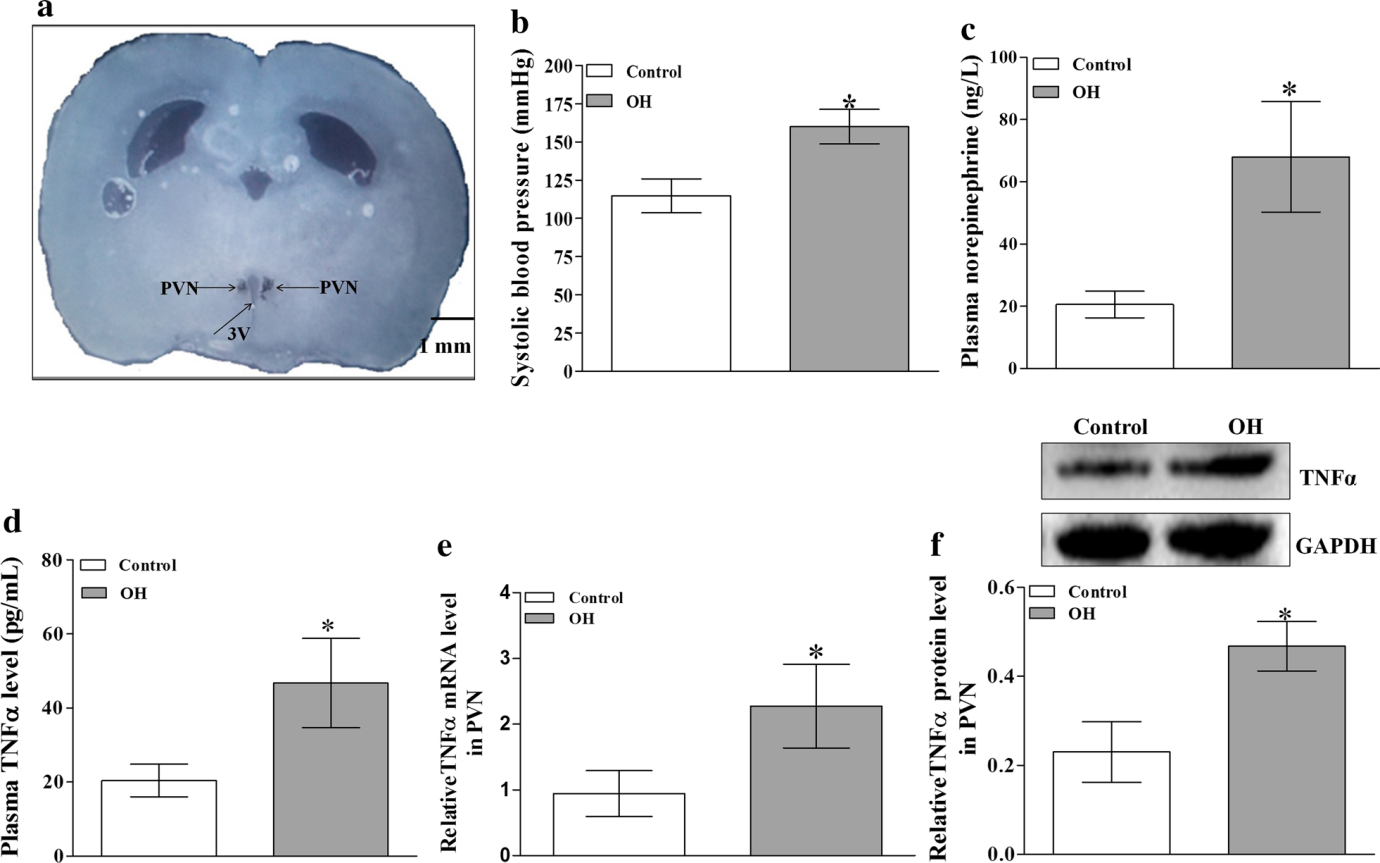
A representative picture of microinjection sites in the PVN evaluated by Evans blue (50 nL) diffusion (**a**); the effect of HFD on the values of systolic blood pressure (SBP) and the plasma levels of norepinephrine (NE) and TNFα (**b**–**d** for each group, n = 7); and the relative values of TNFα protein expression and mRNA levels in the PVN (**e**, **f** for each group, n = 4–5). The values are mean ± SD.*p < 0.05 versus Control. Arrows indicate the microinjection sites. *PVN* paraventricular nucleus, *3V* the third ventricle, *OH* obesity-related hypertension. SBP was measured under conscious state

Stereotaxic coordinates for PVN were 1.8 mm caudal from bregma, 0.4 mm lateral to midline and 7.9 mm ventral to dorsal surface referred to the atlas of the rat brain. To locate the PVN, the rat was fixed in a stereotaxic frame (Chicago, USA), then microinjection volume of 50 nL for each side of PVN was applied by two glass micropipettes (50 μm tip diameter). The bilateral microinjections of chemicals were finished within 1 min. At the end of the experiment, same volume of Evans Blue was injected into the microinjection sites for histological identification of PVN. A representative picture of microinjection sites in the PVN evaluated by Evans blue diffusion was shown in Fig. 1. The data would be excluded from data analysis if microinjection sites were outside the PVN. Total 3 rats in different groups were excluded from data analysis because the microinjection sites were not within the PVN.

### Blood and PVN samples preparation

Each rat was anaesthetized with an overdose of sodium pentobarbital by intraperitoneal injection. For better anesthetic effect, 2% sodium pentobarbital solution was prepared before operation. The rats were anesthetized with the normal dose according to the weight (0.3 mL/100 g). If the animals were not completely anesthetized, we increased the dose about a fifth of the total amount. At this dose, most animals can be completely anesthetized. In general, the anesthesia duration from the beginning to the end is within a few minutes, which generally does not affect the protein expression in the brain. But the protein phosphorylation may be affect by overdose application of sodium pentobarbital, and this needs to be further explored. Blood were collected in heparinized tubes. Plasma samples obtained by centrifugation of heparinized blood for 15 min were used for the estimation of circulating norepinephrine (NE) and TNFα levels. The brain was quickly removed and frozen with liquid nitrogen. Finally, the plasma and brain were stored at − 80 °C until being used. Coronal sections of the brain were obtained with a cryostat microtome (Leica CM1900-1-1, Wetzlar, Hessen, Germany) at the PVN level.

### Measurement of plasma glucose, insulin, cholesterol and triglyceride levels

About 1.5 mL of blood was collected from tail vein. Fasting glucose [using glucose oxidase method and kit from Jiancheng Bioengineering (Nanjing, China)], fasting insulin [using enzyme-linked immunoassay (Elisa) method and kit from RayBiotech, Inc. (Norcross, GA, USA)], cholesterol and triglyceride [using commercial colorimetric assay and kits from Jiancheng Bioengineering (Nanjing, China)] levels in the plasma were measured in Control and OH groups according to the manufacturer’s instructions.

### Measurement of plasma NE and TNFα levels

The levels of NE and TNFα were measured using Elisa method by using kits, and the manufacturer’s instructions were followed. The final solution was read by a microplate reader (ELX800, BioTek, Vermont, USA). The kits for detecting NE and TNFα levels were from R&D systems (Minneapolis, MN, USA).

### Examination of ROS in the PVN

In situ ROS in the PVN were examined using dihydroethidium (DHE) staining. PVN region was achieved from the coronally sectioned (30 μm) brain tissues of rats and it was placed onto chilled slides. The sections were thawed and rehydrated using phosphate-buffered saline, then incubated with DHE (1 μM) in the dark. DHE fluorescence was viewed and imaged under confocal microscopy (Zeiss LSM 510). Moreover, we also detected the content of ROS in the PVN by using the lucigenin-derived chemiluminescence method. Dark-adapted lucigenin can react with ROS causing photon emission which is detected by a luminometer (20/20n, Turner, CA, USA) once every minute for 10 min. The data representing the ROS content is expressed as the MLU per minute per milligram of protein.

### Measurement of NADPH oxidase activity

The activity of PVN NADPH oxidase was determined by the enhanced lucigenin chemiluminescence method [25]. Briefly, NADPH (100 μM) used as a substrate, reacts with NADPH oxidase in the medium to generate ROS which can react with lucigenin (5 μM) to produce light emission. A luminometer (20/20n, Turner, CA, USA) was used to detect the light emission once every minute for 10 min. The data representing the NADPH oxidase activity were expressed as the (MLU) per minute per milligram of protein.

### Measurement of TNFα, NOX2 and NOX4 protein expressions

The protein expressions of TNFα, NOX2 and NOX4 in the PVN were measured as previously reported [26]. Simply, the total PVN protein in the homogenate was extracted and measured. Western blot analysis was carried out by using the rabbit polyclonal antibodies of TNFα (Cell Signaling Technology, Danvers, MA, USA), NOX2, NOX4 (Abcam, Burlingame, CA, USA) and GAPDH (Bioworld Technology, Louis Park, MN, USA) as the primary antibodies. Peroxidase-conjugated goat anti-rabbit secondary antibody (Santa Cruz, CA, USA) was used as secondary antibody. The levels of TNFα, NOX2 and NOX4 protein expression were expressed as the ratio to GAPDH protein, respectively.

### RNA isolation and real-time RT-PCR

The total RNA was extracted from the microdissected PVN by using Tri-Zol reagent (Invitrogen) and reverse transcribed with oligo (dT) and RT. GAPDH was used as housekeeping gene. Real-time RT-PCR was performed in 96-well PCR plates using Bio-Rad PCR Master Mix (the iTaq SYBR Green Supermix with ROX) and the ABI Prism 7900 sequence detection system (Applied Biosystems). The expression levels of TNFα, NOX2 and NOX4 were determined using specific rat primers (TNFα: Forward primer: 5′-CCTTATCTACTCCCAGGTTCTC-3′; Reverse primer: 5′-TTTCTCCTGGTATGAATGGC-3′; NOX2: Forward primer: 5′-CTGCCAGTGTGTCGGAATCT-3′, Reverse primer: AATGGCCGTGTGAAGTGCTA-3′; NOX4: Forward primer: 5′-TGGCCAACGAAGGGGTTAAA-3′, Reverse primer: 5′-CACTGAGAAGTTCAGGGCGT-3′). The values were corrected by GAPDH (Forward primer: 5′-TGATGGGTGTGAACCACGAG-3′, Reverse primer: CCCATAACCCCCACAACACT) and were calculated using the formula *x*  =  2^−(ΔΔCt)^ [28].

### Chemicals

Capsaicin (CAP), PEG-SOD, NADPH (100 μM), lucigenin (5 μM), diethyldithiocarbamate (DETC), Apo, TNFα, R-7050 (10 µM) and pentoxifylline (PTX) were obtained from Sigma Chemical (St. Louis, MO, USA). The doses of CAP (1 nM), PEG-SOD (5 units), DETC (10 nM), Apo (1 nM), TNFα (1 ng) and PTX (30 mg/kg daily ip; in 10% ethanol) were chosen with reference to our preliminary studies and published papers [22, 25, 26].

### Statistical analysis of data

All data illustrated are expressed as mean ± SD. GraphPad Prism v5.00 (GraphPad Software, CA) was used for statistical analyses. Differences in the mean values between two groups were assessed by two-tailed Student *t*-test. One-way and two-way ANOVA were used for data analysis of more than two groups followed by Bonferroni’s post hoc analysis. In all cases, p < 0.05 was considered statistically significant.

## Results

### The effects of HFD consumption on metabolic and anatomic data

After 12 weeks of HFD consumption, plasma insulin, cholesterol, and triglyceride levels, as well as heart weight, body weight and white adipose tissue (WAT) mass were significantly increased in rats with OH (Table 1). The SBP, TNFα and NE (it is often used to evaluate basal SNA) levels in the plasma of rats with OH were much higher than in the Control rats (Fig. 1). These results were almost in line with our previous published results [25]. HFD consumption promoted the increase of plasma TNFα level which may be associated with the enhanced SNA.

**Table 1:** Metabolic parameters and anatomic data in control and OH rats after 12 weeks of diet

Parameters	Control	OH
BW (g)	513 ± 41	627 ± 45*
Plasma glucose (mg/dl)	132 ± 11	143 ± 14
Plasma insulin (ng/ml)	1.61 ± 0.18	2.92 ± 0.29*
Plasma cholesterol (mg/dl)	40.2 ± 4.1	58.9 ± 5.7*
Plasma triglyceride (mg/dl)	62.7 ± 5.9	88.7 ± 8.1*
HW (mg)	1573 ± 92	2148 ± 116*
HW/BW (mg/g)	3.07 ± 0.31	3.43 ± 0.42
Sum of WAT mass (g)	27.5 ± 3.1	56.2 ± 4.8*

Sum of WAT mass includes inguinal, retroperitoneal, epididymal and mesenteric WAT mass. Values are mean ± SEM. n = 10 for each group

*OH* obesity-related hypertensive rats induced by high fat diet, *BW* body weight, *HW* heart weight, *WAT* white adipose tissue

* p < 0.05 versus Control

### HFD consumption promoted the expressions of TNFα mRNA and protein in the PVN

It was reported that TNFα in the PVN contributes to the sympathetic activation [20]. To investigate whether TNFα in the PVN is involved in sympathetic activation and hypertension in OH, we firstly examined the mRNA and protein expressions of TNFα. When compared with the control group, the significant increases of TNFα mRNA (Fig. 1e) and protein levels (Fig. 1f) were found in the PVN of rats with OH. These changes may alter the excitability of neurons in the PVN for enhancing the sympathetic activation.

### PVN microinjection of TNFα enhanced the basal SNA

**Fig. 2:**
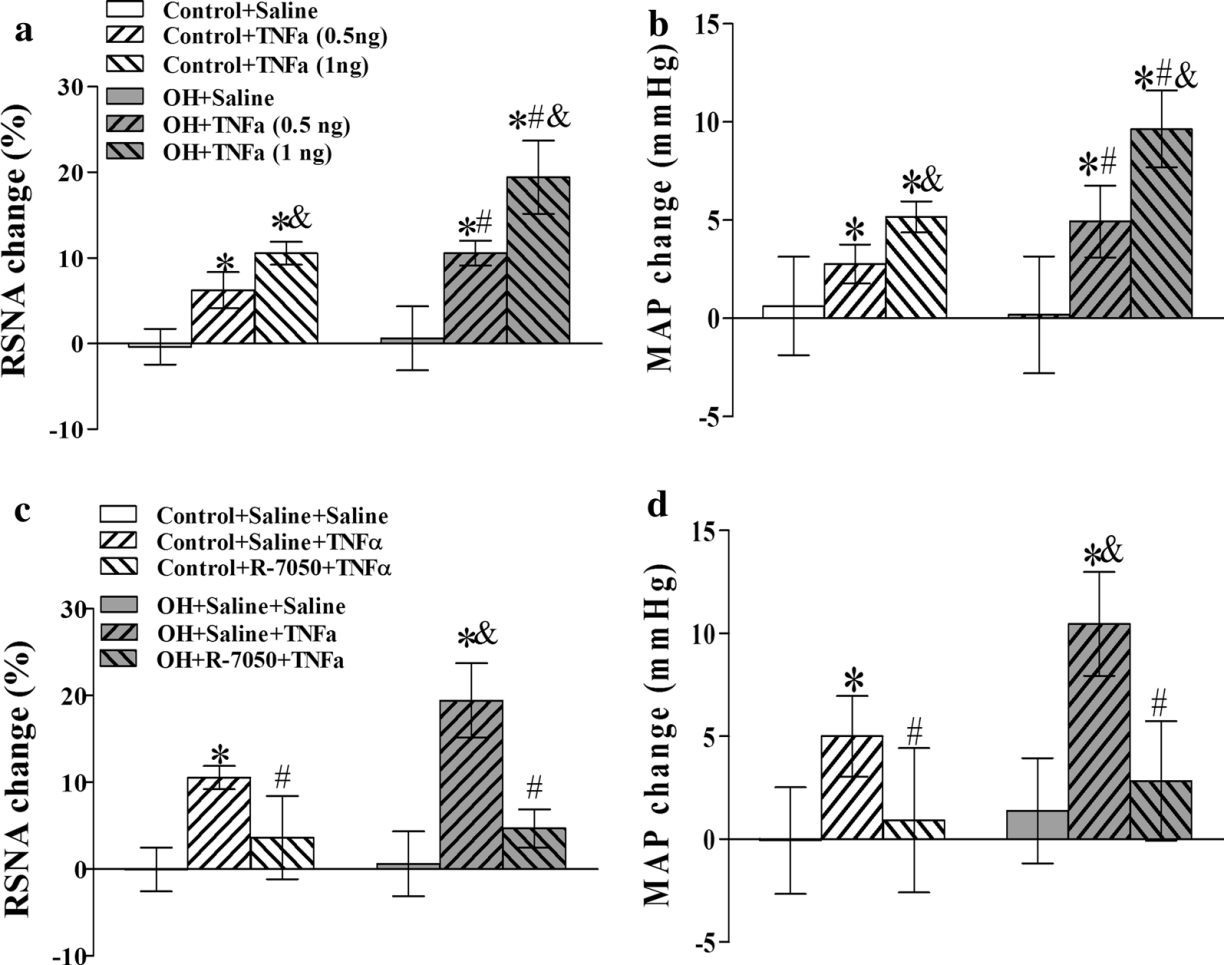
The effects of PVN microinjection of saline and TNFα (0.5 ng or 1 ng) on the baseline RSNA and MAP (**a**, **b**), and TNFα (1 ng) caused SNA response (the changes of RSNA and MAP) to the pretreatment of TNFα receptor antagonist R-7050 in the PVN. The values are mean ± SD. For each group, n = 6–7; *p < 0.05 versus saline (**a**, **b**); ^#^p < 0.05 versus Control (**a**, **b**); ^&^p < 0.05 versus TNFα (0.5 ng **a**, **b**); *p < 0.05 versus Saline + Saline (**c**, **d**); ^&^p < 0.05 versus Control (**c**, **d**); ^#^p < 0.05 versus Saline + TNFα (**c**, **d**). *RSNA* renal sympathetic nerve activity, *MAP* mean artery pressure

The effects of TNFα in the PVN on sympathetic outflow and BP in OH remains unclear, here we investigated the roles of PVN administration of TNFα in RSNA and MAP. Stable baseline values of RSNA and MAP were recorded for 10–15 min, then the saline and TNFα were injected into the PVN, respectively, and the recording was recorded for 90 min. The maximal values of baseline RSNA and MAP were evaluated for 2 min from 30 to 40 min after saline or TNFα microinjection. The peak of the pressor and sympathoexcitory responses due to the microinjection of TNFα into the PVN occured around 15–45 min after the injection, so AAR induction was started 15 min after TNFα infusion. The duration of TNFα’s action in the PVN was around 50–60 min. Acute microinjection of TNFα (1 ng/50 nL) into the bilateral PVN significantly increased basal SNA in the two groups compared with PVN microinjection of saline (p < 0.05 for all comparisons) (Fig. 2a, b), which was significantly inhibited by PVN pretreatment of TNFα receptor antagonist R-7050 (Fig. 2c, d). These results suggested that TNFα via receptor pathway participates in the modulation of SNA.

### TNFα in the PVN strengthened the AAR in rats with OH

**Fig. 3:**
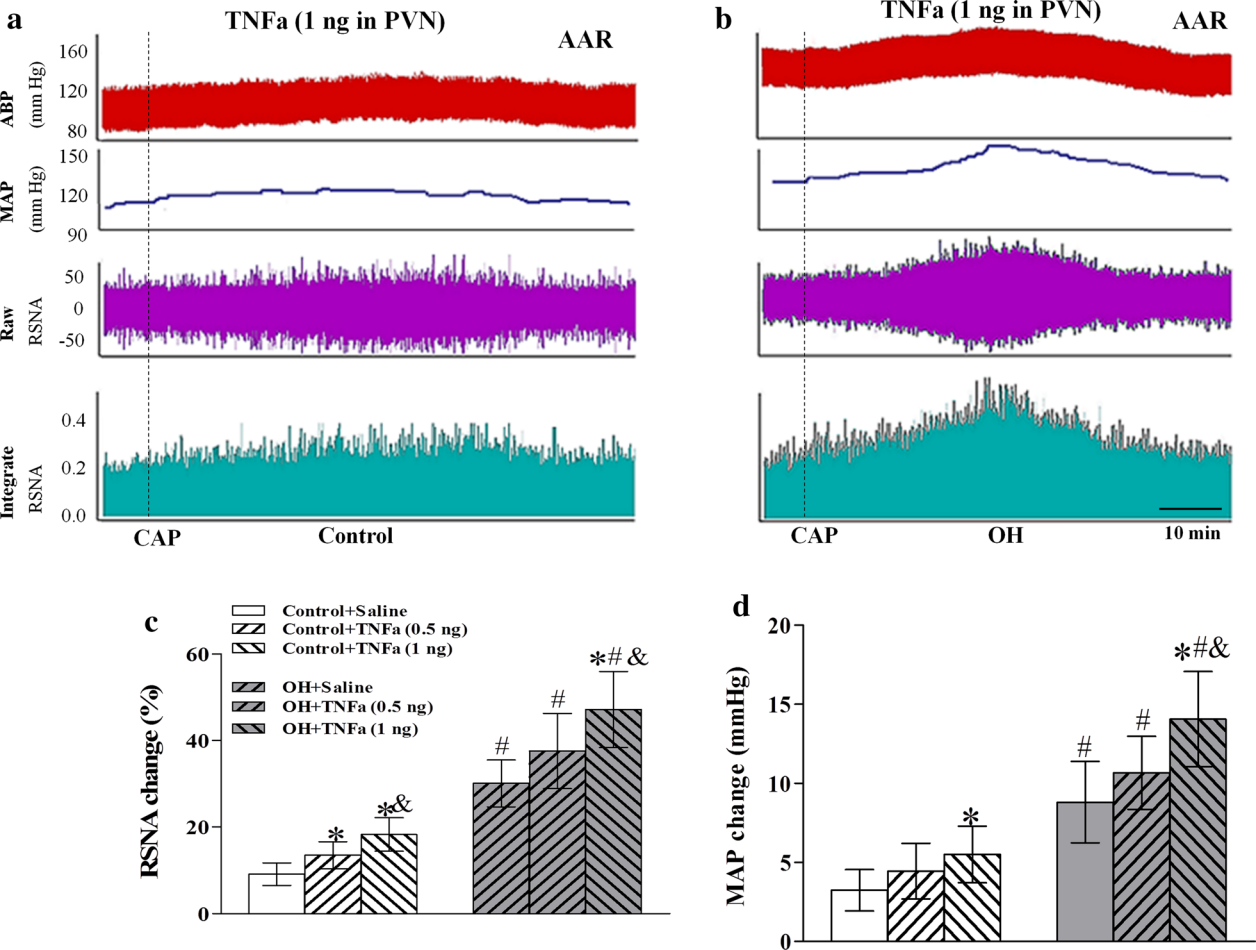
Representative recordings of the capsicin (CAP)-induced AAR are shown by the RSNA and MAP responses to the application of TNFα (1 ng) in the PVN in rats (**a**, **b**); the effect of PVN microinjection of saline and TNFα (0.5 ng or 1 ng) into the PVN on the AAR (**c**, **d**). The values are mean ± SD. For each group, n = 6; *p < 0.05 versus Saline; ^#^p < 0.05 versus Control; ^&^p < 0.05 versus TNFα (0.5 ng). *AAR* adipose afferent reflex

The enhanced AAR contributes to sympathetic activation in diet-induced obese rats or obesity-related hypertensive rats [13]. In this study, the AAR was evaluated by the RSNA and MAP responses to capsaicin (CAP) injection into the right iWAT. CAP is a valuable chemical reagent for studying the function of sensory afferent fibers, and the AAR is induced by CAP (1.0 nM) injection into four sites of the right iWAT at a rate of 4.0 μL/min for 2 min. The AAR was markedly enhanced and TNFα (1 ng/50 nL) injected into PVN significantly increased the AAR in rats with OH when compared with the control rats (p < 0.05, Fig. 3c, d), which indicated that TNFα in the PVN may strengthen the AAR to affect SNA in OH. The representative recordings of the enhancement of AAR caused by TNFα were shown in Fig. 3a, b.

### Pretreatment of R-7050, PEG-SOD or Apo in the PVN inhibited, but DETC augmented the AAR caused by TNFα

**Fig. 4:**
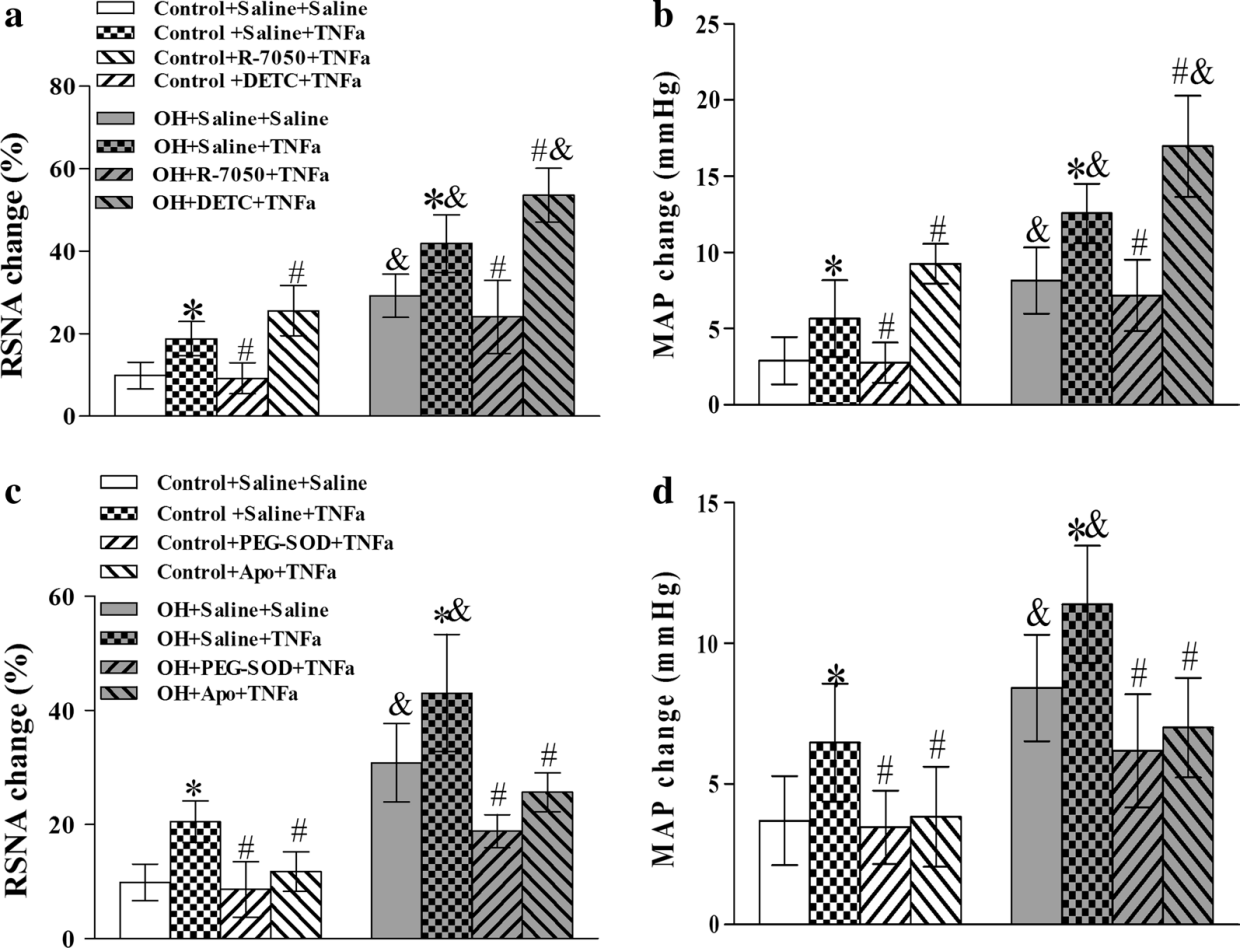
The effects of saline, R-7050 (TNFα receptor antagonist), DETC (superoxide dismutase inhibitor), PEG-SOD (superoxide scavenger) and Apo (NADPH oxidase inhibitor) pretreatment in the PVN on the AAR response to TNFα in the PVN. TNFα (1 ng) was administered 10 min after the pretreatment. The values are mean ± SD. For each group, n = 6–7; *p < 0.05 versus Saline + Saline; ^#^p < 0.05 versus Saline + TNFα (1 ng); ^&^p < 0.05 versus Control

In order to determine whether ROS derived from NADPH oxidase are involved in TNFα-induced enhancement of AAR, we used the ROS scavenger PEG-SOD, a NADPH oxidase inhibitor apocinin (Apo) and a superoxide dismutase inhibitor DETC, respectively, to investigate their effects on the enhanced AAR-induced by TNFα in the PVN. We found that the pretreatment of R-7050, PEG-SOD or Apo in the PVN significantly inhibited the AAR enhancement caused by TNFα in rats with OH, but pretreatment of DETC further strengthened the AAR compared to the single TNFα injection (p < 0.05 for each, Fig. 4a–d). We previously reported that ROS derived from NADPH oxidase in the PVN are involved in the sympathetic activation and hypertension [25]. The results in this study suggested that TNFα via receptor pathway may stimulate the NADPH oxidase activity to increase the production of ROS for promoting the AAR enhancement in OH.

### Pretreatment of R-7050 in the PVN reduced the ROS level and NADPH oxidase activity caused by TNFα

**Fig. 5:**
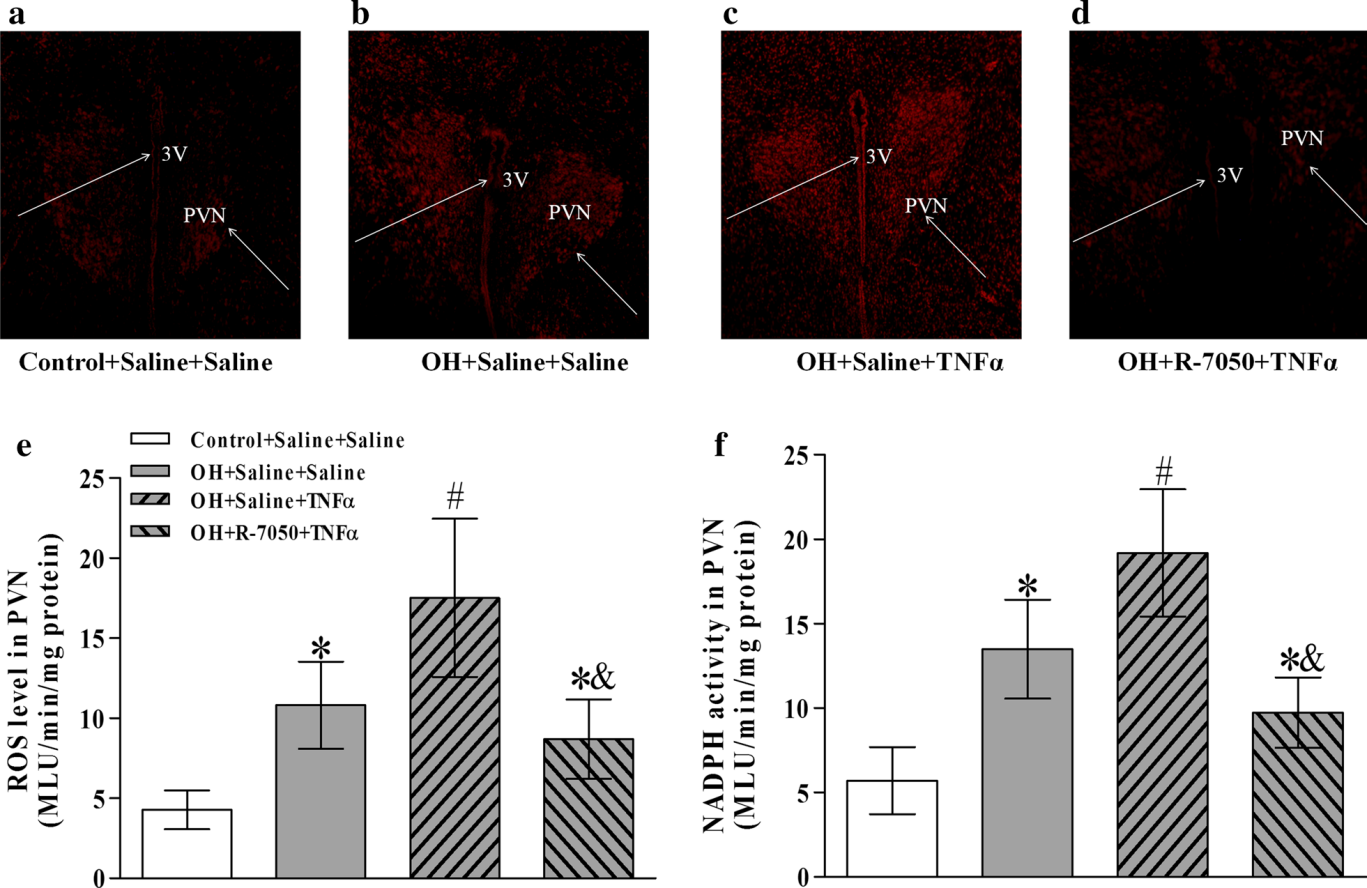
The effects of R-7050 pretreatment on the NADPH oxiase activity (**f**), ROS level (**e**) and the in situ ROS content (**a**–**d**) responses to TNFα in the PVN. In situ ROS was determined with a specific fluorogenic probe by the DHE method (n = 3 for each group). The NADPH oxiase activity was detected by the enhanced lucigenin chemiluminescence method and the ROS level were measured by the lucigenin-derived chemiluminescence method (n = 6 for each group). The values are mean ± SD; *p < 0.05 versus Control + Saline + Saline; ^#^p < 0.05 versus OH + Saline + Saline; ^&^p < 0.05 versus OH + Saline + TNFα. *PVN* paraventricular nucleus, *3V* the third ventricle

For further illustrating TNFα via receptor pathway to stimulate the NADPH oxidase activity and the ROS production, we microinjected TNFα or R-7050 into PVN to determine the NADPH oxidase activity and ROS level. Compared with the microinjection of saline into the PVN, TNFα in the PVN caused significant increases in the ROS level and NADPH oxidase activity in rats with OH, but the pretreatment of R-7050 significantly inhibited the effects of TNFα on them (p < 0.05 for each, Fig. 5e, f). In situ detection of PVN ROS by the dihydroethidium (DHE) method (a superoxide-sensitive dye staining technique) revealed a much higher fluorescent intensity in rats with OH compared with control rats (Fig. 5a–d). These findings verified our hypothesis that TNFα via receptor pathway can stimulate the increases of NADPH oxidase activity and ROS production.

### Chronic pentoxifylline (PTX) treatment on metabolic and anatomic data in rats

After HFD consumption for 12 weeks, PTX (a cytokine blocker, 30 mg/kg, IP) was administrated. The rats with OH continued to be fed HFD for 5 weeks. The results showed that PTX decreased the plasma insulin level when compared with the OH group (Table 2). Although the PTX treatment had a tendency to decrease the heart weight, body weight, and white adipose tissue mass, their changes were not significant (Table 2). The mechanism for PTX decreasing the level of plasma insulin in rats with OH deserves further exploration.

**Table 2:** Effects of pentoxifylline (PTX, a cytokine blocker, 30 mg/kg, IP) treatment on metabolic and anatomic data in rats after HFD consumption for 17 weeks

Parameters	Control	Control + PTX	OH	OH + PTX
BW (g)	559 ± 41	548 ± 43	688 ± 51*	645 ± 49
Plasma glucose (mg/dl)	126 ± 12	122 ± 11	147 ± 16	142 ± 15
Plasma insulin (ng/ml)	1.51 ± 0.3	1.48 ± 0.3	3.12 ± 0.31*	2.09 ± 0.27^**#**^
Plasma cholesterol (mg/dl)	43.1 ± 4.1	42.0 ± 3.9	61.2 ± 6.5*	58.3 ± 5.7
Plasma triglyceride (mg/dl)	66.9 ± 6.6	65.8 ± 7.2	92.6 ± 9.3*	85.2 ± 7.9
HW (mg)	1609 ± 91	1610 ± 112	2218 ± 132*	2021 ± 121
HW/BW (mg/g)	2.91 ± 0.27	2.94 ± 0.35	3.22 ± 0.37	3.13 ± 0.40
Sum of WAT mass (g)	28.5 ± 3.1	27.7 ± 3.3	63.6 ± 5.9*	57.9 ± 4.2

Sum of WAT mass includes inguinal, retroperitoneal, epididymal and mesenteric WAT mass. Values are mean ± SE. n = 10 for each group

*OH* obesity-related hypertension rats induced by high fat diet, *BW* body weight, *HW* heart weight, *WAT* white adipose tissue

* p < 0.05 versus Control; ^**#**^p < 0.05 versus OH

### Chronic PTX treatment decreased sympathetic activity, SBP and AAR

**Fig. 6:**
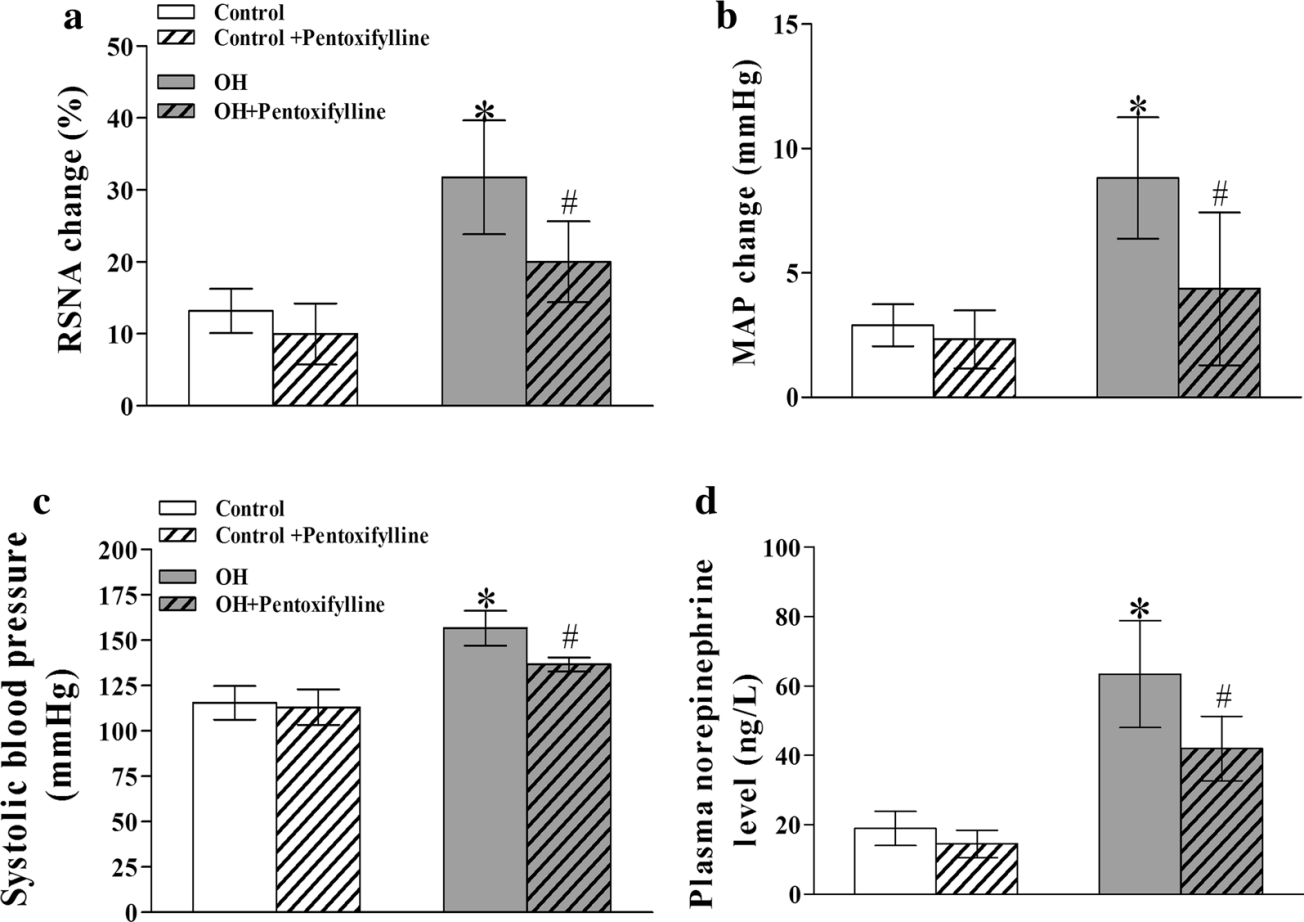
The effects of chronic systemic application of pentoxifylline (PTX, a cytokine blocker, 30 mg/kg, IP) for 5 weeks on the AAR (**a**, **b**), systolic blood pressure (**c**) and plasma norepinephrine levels (**d**) (n = 6–7 for each group) in rats. Values are mean ± SD. *p < 0.05 versus Control; ^#^p < 0.05 versus OH

The AAR, SBP and plasma NE levels were significantly increased in rats with OH, which were significantly decreased by PTX treatment for 5 weeks (p < 0.05 in all cases, Fig. 6a–d). However, the AAR, SBP and NE levels in the control rats treated with PTX were not different from those of control rats. So PTX may inhibit the generation of peripheric TNFα, which further reduces the amount of PVN TNFα coming from periphery being the reason for decreasing the AAR and SNA in rats with OH.

### Chronic PTX treatment lowered ROS and TNFα levels in the PVN, and TNFα level in the plasma

**Fig. 7:**
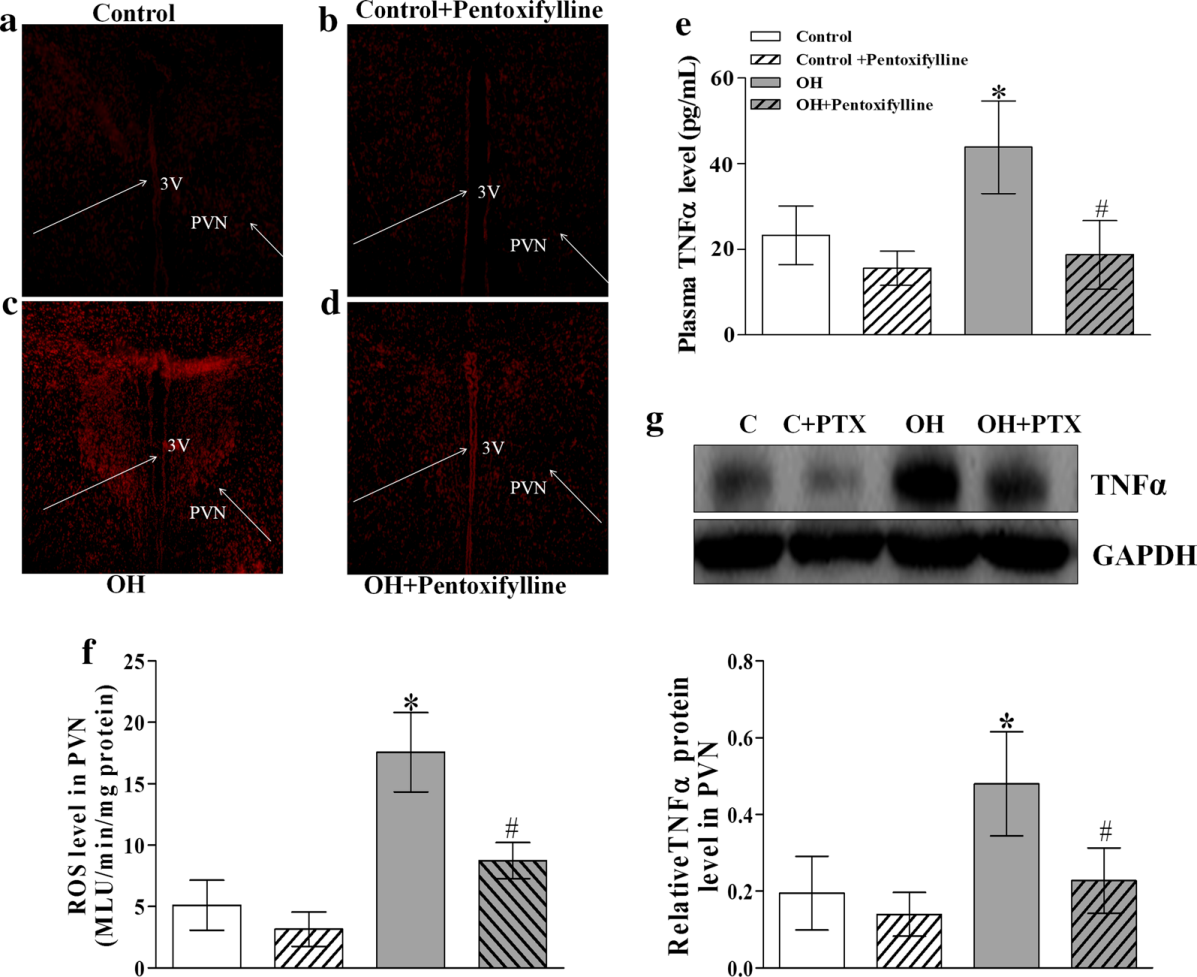
The effects of chronic systemic application of pentoxifylline (PTX, a cytokine blocker, 30 mg/kg, IP) for 5 weeks on the in situ ROS (n = 3 for each group, **a**–**d**), ROS level (n = 6–7 for each group, **f**), protein expression of TNFα in the PVN (n = 3–4 for each group, **g**) and TNFα content in plasma (n = 6–7 for each group, **e**). The values are mean ± SD; *p < 0.05 versus Control; ^#^p < 0.05 versus OH

At the end of 17 weeks, the protein expression of TNF-α and ROS levels in the PVN, and the plasma TNF-α level were significantly increased in rats with OH, which were significantly reduced by PTX chronic treatment. However, PTX in the normal rats had no significant effect on them (p < 0.05 in all cases, Fig. 7e–g). In situ detection of ROS in the PVN illustrated a much stronger fluorescent intensity in rats with OH compared with the control rats (Fig. 7a–d), which was also obviously inhibited by PTX treatment. These findings indicated that the inhibition of cytokines generation can reduce ROS production in the PVN of rats with OH.

### Chronic PTX treatment reduced the mRNA and protein expressions of NADPH oxidase isoforms (NOX2 and NOX4) in the PVN

**Fig. 8:**
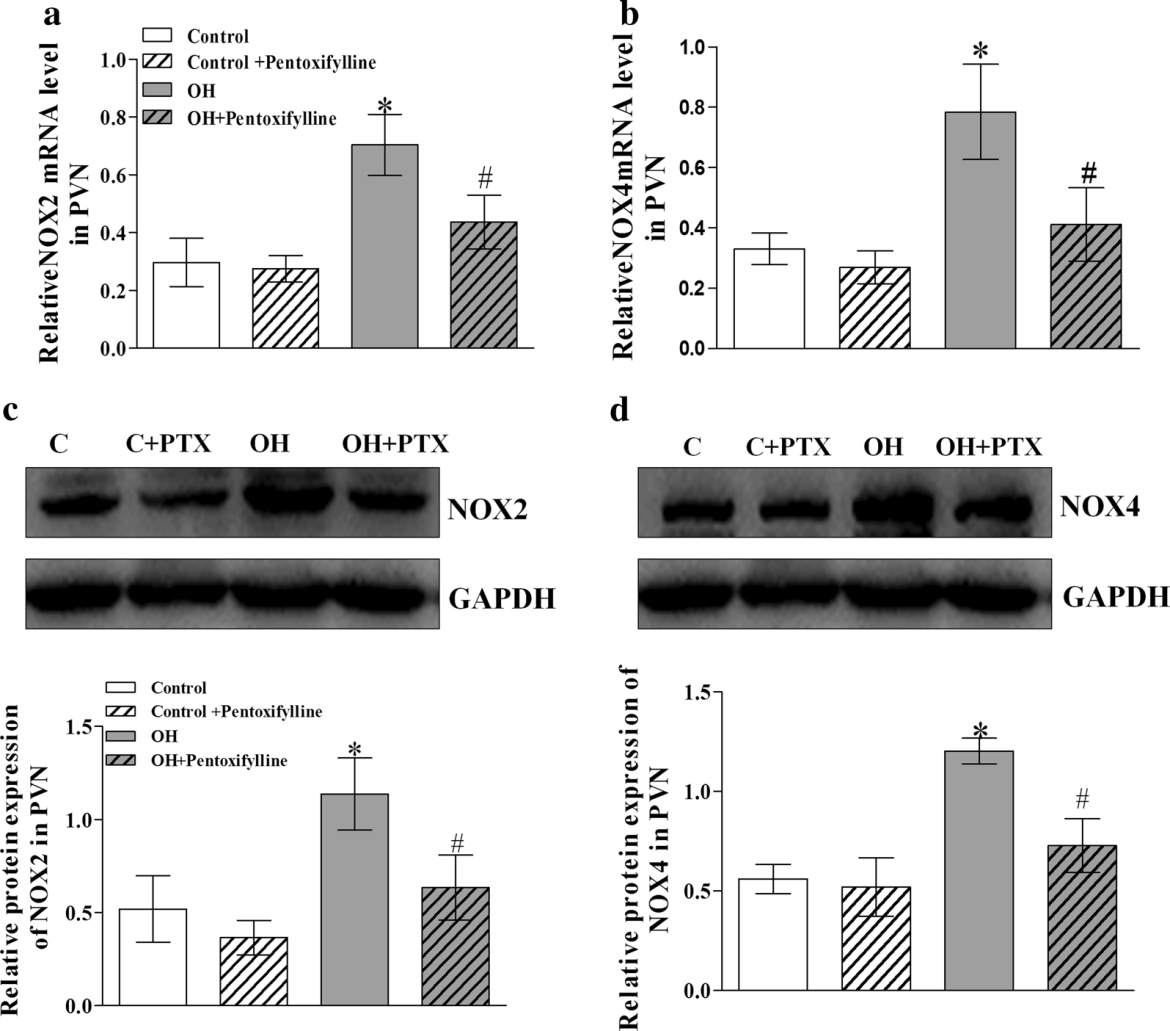
The effects of chronic systemic application of pentoxifylline (PTX, a cytokine blocker, 30 mg/kg, IP) for 5 weeks on the mRNA (**a**, **b**) and protein (**c**, **d**) expressions of the NOX2 and NOX4 isoforms of NADPH oxidase. The values are mean ± SD; *p < 0.05 versus Control; ^#^p < 0.05 versus OH

The levels of mRNA and protein expression of NADPH oxidase isoforms (NOX2 and NOX4) in the PVN were significantly upregulated in rats with OH when compared with the control rats. Similarly, the elevated mRNA and protein levels of NOX2 and NOX4 in the PVN were effectively inhibited in PTX-treated rats with OH (p < 0.05 in all cases, Fig. 8a–d). The findings revealed that the inhibition of cytokines production suppressed the mRNA and protein expressions of NADPH oxidase isoforms (NOX2 and NOX4) in the PVN of rats with OH.

## Discussion

The present study indicates HFD not only induced adiposity which was associated with an enhanced AAR and sympathoexcitation, as reflected by the increased plasma NE level and BP, but also caused an elevated concentration of circulating and PVN inflammatory cytokine TNFα. Acute microinjection of TNFα into the PVN not only increased NADPH oxidase activity and ROS levels, but also caused significant increase in the AAR, which was inhibited by ROS scavenger PEG-SOD or NADPH oxidase inhibitor Apo in rats with OH. Chronic systematic suppression of TNFα with PTX during the HFD period blunted TNFα production and attenuated the enhanced the AAR, SNA and hypertension in rats with OH. PTX also decreased the mRNA and protein expressions of NADPH oxidase subunits and ROS level in the PVN of HFD-fed rats. Together, these findings suggested that chronic HFD consumption causes an increase of inflammatory cytokine TNFα in the PVN which appears to promote the increase of ROS, initiate the elevation of AAR, and further result in the elevation of SNA and BP.

Elevated TNFα levels in the PVN can evoke sympathoexcitation [24, 25]. TNFα blockade attenuated NE levels in the hypothalamus, suggesting a major intermediary role for TNFα in sympathoexcitation [21]. In this study, HFD induced the increases in TNFα protein and mRNA levels in the PVN. Acute microinjection of TNFα into the PVN caused the significant increases in the AAR, SNA and BP, which was inhibited by TNFα receptor antagonist used in the PVN of rats with OH. This suggests that the increased TNFα level in the PVN of rats with OH can promote the enhancement of AAR involving sympathoexcitation and hypertension. It is also possible that TNFα produced from other regions of the brain, or as a blood-borne cytokine crosses the circumventricular organs that lack a blood–brain barrier (BBB), diffused into the region of the PVN and contributed to the AAR enhancement. Moreover, OH-induced AAR increase was attenuated by TNF blockade by using PTX systemically, a cytokine inhibitor, suggesting a role for TNFα in sympathoexcitation in OH.

ROS can act as key modulators of increased neuronal activity in the PVN of rats with hypertension, heart failure or metabolic syndrome [29–32]. More importantly, ROS in the PVN can modulate AAR [25]. In our study, administration of TNFα into PVN increased the level of ROS in the PVN, and ROS clearance led to decreased AAR and sympathoexcitation caused by TNFα. TNFα receptor antagonist R-7050 in the PVN also inhibited the ROS generation. Moreover, systemic administration of anticytokine agent PTX resulted in reduced AAR, SNA and ROS production in the PVN of rats with OH. Therefore, it is possible that the increased TNFα observed in the PVN can trigger ROS production that lead to AAR enhancement and sympathoexcitation in OH.

The NADPH oxidases are one of the major sources of ROS in the PVN, and its subunits NOX2 and NOX4 in the PVN have been implicated in the development of oxidative stress and sympathoexcitation [33–35]. Furthermore, in the PVN, they were also involved in the sympathetic overactivation in rats with OH [25]. It is important to point out that in the PVN, TNFα can regulate NADPH oxidase activity to affect ROS generation [23]. TNFα-initiated elevation of AAR in the PVN may be associated with NADPH oxidase dependent generation of ROS. In this study, TNFα administration into the PVN can induce the increase of NADPH oxidase activity in rats with OH, which can be inhibited by a TNFα receptor antagonist. Moreover, NADPH oxidase inhibitor APO almost abolished the increases of AAR and SNA caused by TNFα. Furthermore, the mRNA and protein expression levels of catalytic subunits of NADPH oxidase NOX2 and NOX4 and ROS content in the PVN were elevated in rats with OH, which were significantly reduced by systemic PTX application. Taken together, these results raise the possibility that TNFα in the PVN induces AAR enhancement in OH, which was mediated by the increased NADPH oxidase-dependent ROS generation.

## Conclusions

The current study indicates that feeding a high-fat diet induces much more inflammatory cytokine TNFα production in the PVN, which activates NADPH oxidase activity to increase ROS generation for enhancing AAR, SNA and hypertension in rats with OH. Therapeutic strategies to reduce the PVN inflammatory cytokine TNFα may play an effective role in preventing the elevation of SNA and BP in OH.

## Data Availability

The datasets used and/or analyzed in this study will be made available by the authors on reasonable request.

## Abbreviations

AAR: adipose afferent reflex
SNA: sympathetic nerve activity
BP: blood pressure
PVN: paraventricular nucleus
TNFα: tumor necrosis factor alpha
OH: obesity-related hypertension
RSNA: renal sympathetic nerve activity
MAP: mean arterial pressure
CAP: capsaicin
SBP: systolic blood pressure
NE: norepinephrine
ROS: reactive oxygen species
DETC: diethyldithiocarbamate
Apo: apocynin
PTX: pentoxifylline
WAT: white adipose tissue
PICs: proinflammatory cytokines
CHF: chronic heart failure
HFD: high-fat diet
BW: body weight
iWAT: inguinal WAT
DHE: dihydroethidium
GAPDH: glyceraldehyde-3-phosphate dehydrogenase
